# Developing modern primary care nursing in North Macedonia

**DOI:** 10.1017/S1463423623000348

**Published:** 2023-08-14

**Authors:** Rosamund Bryar, Peter P. Groenewegen, Mireia Sánchez Martínez, Cris Scotter

**Affiliations:** 1 Professor Emerita Community and Primary Care Nursing, City, University of London, London, UK; 2 Netherlands Institute for Health Services Research (NIVEL), Utrecht, The Netherlands; 3 Independent Consultant, Barcelona, Spain; 4 WHO Policy Advisor (Regional Office for Europe), Copenhagen, Denmark

**Keywords:** continuing professional development, North Macedonia, primary care nursing

## Abstract

**Background:**

Nurses have the potential to make a real impact on the health and well-being of people and populations and contribute to the realisation of delivery of Universal Health Coverage. However, in many parts of the world, the education and practice of nursing and nurses’ position in health care and society are restricted by a range of social, cultural, economic and political factors. In North Macedonia, the Ministry of Health in partnership with the WHO Country Office launched a primary healthcare strategy supporting the development of nurses in primary care to fulfil their full scope of service.

**Aims:**

To present information on the education, practice and position of nursing, in particular primary care nursing, in North Macedonia and to describe the ongoing initiatives to support the further development of nursing.

**Approach:**

Background documents reviewed, and visits to healthcare settings, organisations, interviews with individuals and groups and workshops undertaken in 2019–2020.

**Findings:**

Three key areas of development were identified: education of nurses, their service delivery and practice in primary care, and their position in health care and society, all underpinned by the need for workforce planning. The findings formed the basis of a 10-year plan: Making Change Happen: The Nursing and Midwifery Development Roadmap.

**Developments:**

To support the proposed primary care pilots, during the 2020/2021 COVID-19 pandemic, an on-line modular programme for primary care nurses was developed and delivered with the support of members drawn from The National Working Group for Moving Primary Care Nursing Forward in North Macedonia. Further work is planned to develop initial nurse education and to pilot changes in primary care.

**Conclusions:**

The launch of the primary healthcare strategy stimulated initiatives to improve the education, position and practice of primary care nursing. The COVID-19 pandemic required flexibility and changes to the original plans.

## Introduction

This paper describes the development and early stages of the implementation of a 10-year Roadmap to support the development of nursing in North Macedonia. The strategy (Roadmap) development was informed by reference to national and international policy and three assessment visits to the country in 2019–2020. Following discussion of the policy context, we discuss the information collected during the assessment visits and outline the elements of the Roadmap. Implementation of the change process informed by the Roadmap commenced in 2019 and actions implemented, including training to support the development of primary care nurses, are discussed. This paper concludes with reference to ongoing work to support the development of the education, practice, leadership and social position of nurses and midwives in the country.

Nurses are the largest profession in healthcare systems, and they play a key role in health services delivery and prevention (WHO, 2016a; 2021). Investing in nurses improves patient experiences and health outcomes and enables doctors to practice to the full extent of their competence (Martinez-Gonzalez *et al.*, 2015; Lovink *et al.*, 2017; Laurant *et al.*, 2018). Nurses also play an important role in transforming healthcare systems (Groenewegen, 2008). The WHO *Global Strategy on Human Resources for Health* (WHO, 2016b) emphasises the need for all countries to assure ‘effective coverage’ (Campbell *et al.*, 2013) by addressing the ‘availability’, ‘accessibility’, ‘acceptability’ and ‘quality’ (as defined by WHO, 2016b) of their healthcare workforce to enable achievement of Universal Health Coverage, attainment of the Sustainable Development Goals and well-being of populations. The strategy recognises that countries face different challenges in developing an effective healthcare workforce dependent on the context in which the country is operating.

The International Council of Nurses (ICN) defines nursing as follows:

‘Nursing encompasses autonomous and collaborative care of individuals of all ages, families, groups and communities, sick or well and in all settings. Nursing includes the promotion of health, prevention of illness, and the care of ill, disabled and dying people. Advocacy, promotion of a safe environment, research, participation in shaping health policy and in patient and health systems management, and education are also key nursing roles’. (ICN, 2002)

This definition indicates the wide-ranging role that nurses play or could play, addressing the emerging health needs of the population. The WHO (2021) document sets out the strategic direction for nursing and midwifery and also identifies the limitations that nurses and midwives may face in different parts of the world in actually fulfilling the ICN definition. Regulation often prevents them from working to the full extent of their education and training, lack of international recognition of their education restricts mobility and they are often not allowed to share information with other health professionals (APPG, 2016: 52; WHO, 2021: 13).

The education, practice and position of nurses differ markedly between countries. Education varies from vocational training at different levels to university programmes up to PhD level. The scope of practice in some countries, for example, in Ireland, Sweden and New Zealand, is broad and nurses have an autonomous professional position, including the right to prescribe medicines (Kroezen *et al.*, 2012). In other countries, for example, in Armenia, Azerbaijan and Ukraine, nurses’ roles are much more restricted, with a focus on providing support to physicians and administration (Sahakyan *et al.*, 2020). In many countries, nursing practice is restricted to those who are registered on an official register, with the right to the title of Registered Nurse. The position of nurses reflects their social status, and clinical, economic and organisational autonomy. As such this influences their labour market position and opportunities to influence their position through influence on policy making.

WHO’s *Global Strategy on Human Resources for Health* emphasises the importance of improving educational processes and labour market dynamics to create a workforce that can provide integrated, person-centred care with universal coverage of the population (WHO, 2016b). The findings of a scoping review of literature concerning the global nurse workforce shortage (Drennan and Ross, 2019) provide evidence supporting the multiple policy areas that need to be addressed to improve supply, retention and productivity of nurses at the macro/meso and micro levels in a country. These include, for example, promoting a positive image of nurses, establishing educational standards, establishing regulatory bodies and having effective human resource policies (see Drennan and Ross, 2019: Table 3: 8–9). These requirements are reinforced in the work of the European Federation of Nurses Associations (EFN) which has identified three categories of nurse: general care nurses, specialist nurses and advanced nurse practitioners supported by healthcare assistants and the education, competencies and qualifications required for each of these roles (EFN, 2017a; 2017b). In this paper, we have adopted the broader definition of nurse used in the data collected in the WHO report: *State of the world’s nurses 2020: investing in jobs, education and leadership* (WHO, 2020) in which the collection of the data on the numbers of nurses in each country was based on the definition of nurse in a particular country. As discussed above, in some countries, the nursing scope of practice is restricted, and the work of EFN and other bodies provides support in enabling nurses in these countries to work towards the full recognition and practice of the role of professional nurse.

## Background

Box 1 briefly describes the country, North Macedonia, and its healthcare system.

Box 1. North Macedonia and its Healthcare System

**Programme Structure**

North Macedonia is the southern-most of the countries which were part of the Former Yugoslavia. It is situated on the Balkan, neighbouring to Greece in the South, Bulgaria in the East, Serbia in the North and Albania in the West. It is a small country in terms of population, with 2,083,380 million inhabitants in 2020 (The World Bank, 2020). It is a multiethnic country in terms of population groups, languages and religion. It is an upper middle-income country in the classification of the International Monetary Fund with a gross domestic product per head of 15,709 US purchasing power parity dollars.

Following the separation of countries from the Republic of Yugoslavia in 1991, the separate countries started to reform their (primary) healthcare systems. Some kept the strong emphasis on public health centres, while others created a mix of private provision of primary care and health centres but in North Macedonia the role of the health centres was strongly reduced (Klancar and Svab, 2014). The healthcare system of North Macedonia is based on a social health insurance system with a single Health Insurance Fund (HIF) as purchaser of care in the private sector (Milevska Kostova *et al.*, 2017). Insurance coverage is estimated to be around 89% of the population in 2018. The extent of the benefits package is wide and covers most of the necessary care. Health expenditure is relatively low, both per head of the population and as a percentage of gross domestic product. Public spending on health is relatively low as a share of GDP: 4% of GDP in 2018, compared to an average of 6% for European Union (EU) countries (WHO, 2020). Out-of-pocket payments are high by EU standards. In 2018, the out-of-pocket payment share of current spending on health was 42%, well above the EU average of 22% (WHO, 2020) and is largely driven by out-of-pocket payments for outpatient medicines and informal services payments.

Primary care in North Macedonia is characterised by a limited scope of practice of physicians leading to high referral and avoidable hospital admission rates. General practitioners (GPs) and family medicine (FMs) specialists were privatised in the years 2004–2007 and some rent their premises from their former employers, the health centres. The majority, 68%, (in 2018) work in separate single-handed practices, even when working under the same roof in a health centre. They are required to employ a nurse to be designated as a general practice but do not have any practice management/administrative support (WHO Regional Office for Europe, 2019). GPs have completed initial medical education programmes but no specialist GP education. FMs have additional education through a 3–4-year specialist programme. The role of nurses in primary care is severely limited with no definition of their competencies and the scope of their service. Their role is concerned largely with administrative processes in the GP practice (Martinez and Sánchez Martínez, 2018; WHO, 2018). While nurses in the country may be categorised as general care nurses (EFN, 2017a, 2017b) their: ‘… non-standardized, sub-optimal education …’ (Atanasova and Tawilah, 2021: 6) raises concerns about their basic education, a priority area for the EFN. The reports (Martinez and Sánchez Martínez, 2018; Marti *et al.*, 2020) provided proposals to expand the scope of practice of primary care nurses but also of GPs/FMs, to integrate care both within the primary care sector and with secondary care and to introduce quality indicators.

These proposals echo the Declaration of Astana (WHO, 2018: 5), reiterating the centrality of primary health care (PHC), enshrined in the 1978 Declaration of Alma-Ata (WHO, 1978), in the achievement of population health and well-being (WHO, 2015). Central to the Declaration of Astana is the need to develop the healthcare workforce including the nursing and midwifery workforce. In response to the Declaration of Astana, the Ministry of Health (MoH) of North Macedonia developed, together with WHO and a wide range of partners in the country, a new PHC-based vision for health care in the country. It proposes a change towards an integrated, people-centred, preventive healthcare system with a focus on primary care (Ministry of Health, 2019). The North Macedonian PHC Strategy emphasises that nurses are critical to the well-being of the population and urges the need to enable nurses to fulfil their full potential, for example, through continuous professional education, reduction in the administrative burden in primary care and by licensing and regulation (Ministry of Health, 2019). Development of primary care is a precursor to the achievement of the wider vision of PHC (Bryar, 2000).

## Approach to assess nursing and midwifery in North Macedonia

In 2019, the authors (RB, PG, MSM) were invited by the MoH in collaboration with the WHO Country Office and WHO Regional Office for Europe (CS) to undertake an assessment of the situation of nursing and midwifery in North Macedonia with a focus on the role of nurses in primary care, building on a previous mission (Martinez and Sánchez Martínez, 2018). Subsequently, two more visits were made to the country by one or more of the authors, and a nursing development Roadmap was initiated.

In undertaking the three visits to the country in 2019 and 2020, reported in this paper, the focus moved from a wide collection of information on the issues concerning nursing and midwifery to a focus on the development of the role of nurses in PHC and their role in a proposed pilot study of a new model of working in PHC. The authors between them have expertise in PHC, nursing, midwifery, policy analysis, workforce planning, public health and education. In addition, on the first visit we were joined by a national from North Macedonia with expertise in public health and the third visit was led by a wider team including international experts with PHC, policy and public health expertise.

The collection of information to meet the objectives of each visit (see below) on all three occasions was informed by reading background documents. Information was collected by making site visits. Meetings were held with GPs/FMs and primary care nurses, patronage nurses (public health nurses who provide care for families with young children and for people aged over 60 years (Kisman and Donev, 2007)), nursing students and their schoolteachers, a patient group representative and university representatives. Central meetings were attended by representatives including from the Chambers of Doctors and Health Professions, the Association of Nurses, Midwives and Technicians, Ministries of Health and Education and Science. Presentations were made by Macedonian representatives and members of the visiting teams. Throughout the visits, meetings were held to discuss and validate findings with the WHO Country Office.

To enable the gathering of a wide range of information in the first visit in early 2019, the initial objectives of the assessment were to:

1. Assess the current situation of Nursing and Midwifery and sustainability in areas of regulation, leadership development, planning and educating the workforce, policy and healthcare delivery as well as payment mechanisms.
2. Explore the possibility of advanced nursing practice at PHC and/or improving training for community nurses – patronage system.
3. Identify measures to be addressed in areas of regulation, leadership development, development of national strategic planning, etc.
4. Advocate for development of new roles, such as family health nursing, community nursing and advanced nursing practice.

For the second visit, the objectives had become more focused, drawing on the findings from the first visit and using the Roadmap developed after the first visit, and included an educational programme to support pilot projects in primary care:

1. Diagnostics and agreements about scope and standards of nursing practice in general and in primary care involving a number of essential stakeholders and decision makers.
2. Feasibility and development of a train-the-trainers’ programme for nurses in primary care and identification of a training institution to be involved from the inception of the programme.
3. Identification and preparation of the primary care pilot development sites.
4. Outline action plan for 2020 and the external expert support needed.

Having identified during the second visit the additional training needs for nurses to take on a health promotion and management of chronic diseases role, the nursing input into the third visit (January 2020 by one of the authors (RB)) was concerned with the integration of this role into the proposed PHC pilots. The aim of the third visit by a wider group of PHC academics and policy professionals was focused on the PC pilots. The central purpose of this visit was to design a PHC model tailored to the North Macedonian context and an operational plan to implement it in two demonstration sites.

Specific objectives were to:

1. Make a rapid assessment of the demonstration sites and evaluate their readiness to proceed.
2. Develop a rationale to justify the operation mode and funding from the Government to sustain the demonstration sites.
3. Discuss and agree with the demonstration sites on implementation plans.
4. Plan for developing integrated practices in those centres.
5. Develop a monitoring and evaluation framework for the demonstration sites (Marti *et al.*, 2020).

## Findings

**Figure 1.**
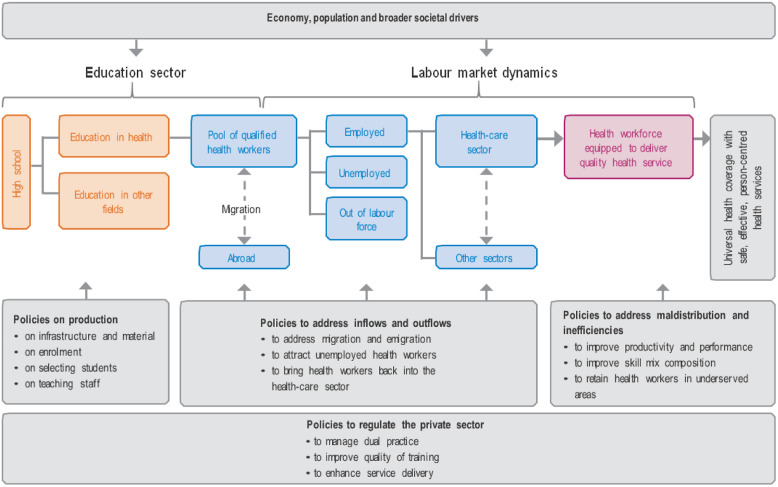
Health labour market context diagram. (*Source:* Fig. 2: Policy levers to shape health labour markets from: Global strategy on human resources for health: Workforce 2030. WHO, Geneva, 2016b: 13).

The information we collected during the first visit reflected the view demonstrated in the WHO’s Global Strategy on Human Resources for Health (WHO, 2016b), illustrated in the Health Labour Market Context Diagram (see Fig. 1). We use this diagram to highlight the interconnectedness of policies which impact on the development and maintenance of an effective healthcare workforce and the relationship between these policies and the wider policy context (Buchan *et al.*, 2019). The educational system (left side of the diagram) prepares students for work in health care and they are added to the pool of healthcare workers. Part of this pool actually works in the healthcare sector and, through their educational preparation, contributes to the delivery of good quality services. This in its turn contributes to the performance of the healthcare system. The diagram shows that attrition takes place at several points, e.g., trained healthcare workers who migrate abroad, and the policies that may influence the educational system, the in- and outflow of healthcare workers and the service provision.

In North Macedonia, nurses form the largest group of healthcare practitioners: in 2019 there were 9,943 licensed nurses, 6,468 licensed physicians and 1,003 licensed midwives (Ministry of Health, 2021). However, these figures also show the imbalance in the proportion of nurses to doctors with approximately 1.3 nurses to 1 doctor. The ratio of nurses to doctors compares to that in Chile, Turkey and Mexico with, in 2015, less than 1.2 nurses to each doctor as compared to other OECD countries where there were between 2 and 5 nurses per doctor in Japan, Finland and Denmark (OECD, 2017; Groenewegen *et al.*, 2019a).

During the first visit, we were exposed to a large number of ideas and issues, and we developed an understanding of the challenges faced by the healthcare system and by nurses and midwives. In line with the Health Labour Market Framework (Fig. 1) and the evidence collected, the proposed actions have been categorised into three areas or pillars:

1. **Education of nurses:** This concerns the policies on ‘production’ of the health workforce (the left part of the diagram in Fig. 1).
2. **Position in health care and society**: In the Health Labour Market Framework, focus is on the labour market position that may influence the attractiveness to nurses and midwives to work in the North Macedonian healthcare system which is important in relation to the migration of nurses from North Macedonia to EU countries (the middle, blue part of Fig. 1).
3. **Practice of service delivery**: This concerns policies to improve **service delivery/practice** by nurses in order to contribute to integrated, person-centred care, focusing here on the role of nurses in primary care (the right side of the diagram in Fig. 1).

Workforce planning is of crucial importance to support developments in these three main areas (WHO, 2016b).

### The education of nurses

The initial education of nurses takes place in secondary high schools. Vocational nursing degrees are offered at three universities as well as specialist programmes which are also offered in a higher training institute for nurses. In each of the high schools we visited, we met with members of the nurse teaching team, were shown the theory and clinical teaching rooms and in one school met with second-year students.

Young people who have completed 9 years of primary education can progress to high school education. All students aiming to work in health care undertake the 4-year Technical Vocational Education programme in nursing. On graduation at age 18, they gain a diploma and can then undertake a 6-month internship in a hospital and take the state examination to qualify as a nurse. However, the vast majority (80%-90%) of students who undertake the nursing vocational programme at high school go on to study medicine, dentistry or another subject at University and do not, therefore, join the nursing workforce.

A very small number of young people, on completion of the high school diploma, will go on to undertake the 3-year professional studies (vocational) degree in nursing, graduating with the title graduate nurse. Following graduation, these people have to undertake a 10-month internship before taking the state examination. The examination is administered by the MoH. Some graduate nurses will then undertake a further year of specialised professional studies graduating with the title of nurse in the particular specialty, for example, intensive care, oncology or family and patronage care. In addition, a number of nurses will pursue these programmes later in their careers enabling them to become head nurses.

Teaching in the high schools is undertaken by subject specialists, for example, biologists, and qualified doctors, employed in the high schools, undertake the teaching of all theory related to nursing. Nurse teachers teach practical nursing skills and supervise students when they work in the clinical areas. This division also applies to the delivery of the university programmes. Nursing is classified as a vocational degree as opposed to an academic degree. There is no progression allowed from a vocational degree to higher degrees including Master’s and PhDs. Lecturers in universities are required to have an academic degree which means that nurses cannot progress to be lecturers (unless they have an academic degree in another subject). The challenges to nurse education are summarised in Box 2.

Box 2. Summary of Nurse Education Challenges in 2019

- Current high school nursing programmes prepare students for university programmes in medicine and other health-related topics rather than being focused on preparing nurses to work in the health system as nurses
- Nurses and midwives are prevented from teaching theoretical subjects due to two laws: one which requires doctors to teach theoretical subjects and a second which limits nursing and midwifery degrees to 3-year vocational programmes
- Programmes weighted towards theory with less time for clinical practice
- Programmes do not meet the EU Directive on nurse education
- Lack of continuous professional development (CPD) provision
- Lack of standards for re-registration including the requirement for CPD

In line with the 10-year Roadmap, the WHO Country Office commissioned a team from Coventry University (UK) to work with a WHO team to undertake and report on a feasibility study of the educational changes needed to reform nurse education in the country (WHO Regional Office for Europe, 2021). Plans are being put in place to develop nurse educators to support the professionalisation of nurse education in the country.

### The position of nurses in health care and society

The position of nurses and midwives in society is weak. There are several reasons for this. The ‘public’ does not see nursing and midwifery as professions. The income of nurses is low. The high school education in nursing is seen by the large majority of students as a step towards university study in medicine or another discipline but not towards becoming a nursing professional.

Characteristic for the position of professions is that they apply theoretical knowledge in the treatment of individual cases, and they have clinical autonomy in coming to their decisions. As discussed above, theoretical knowledge in nurse education is provided by medical doctors. Nurses therefore lack an important characteristic of professions. Moreover, nurses, in particular in primary care, lack clinical autonomy. Autonomy over the content of work is another key characteristic of professions and an important determinant of job satisfaction and retention of nurses (Maurits, 2019).

Differences in specialised education of nurses are not translated into differences in payment, according to the nurses we met during our site visits (see also Milevska Kostova *et al.*, 2017). In general, the economic position of nurses, especially in private primary care and public hospitals, is weak. As a consequence of all this, many nurses migrate to EU countries after finishing their education.

Regulation of nursing is also not developed. Other health practitioners, for example, doctors and dentists, are regulated by Chambers. The Chambers provide professional oversight of the quality of the education and practice of the profession, maintain registers and manage disciplinary processes. In 2019, a Chamber for Health Professions was established, and nurses and midwives were included in this Chamber. However, the disproportionate numbers of nurses and midwives compared to other health professions argue for a separate Chamber for nurses and midwives. Box 3 summarises the main findings regarding the position of nurses in 2019.

Box 3. Summary of Position of Nurses in 2019

- There is no Nursing and Midwifery Chamber that can serve as a focal point for professional development and influence on policy
- There is no registration and licensing of nurses and midwives
- Nurses and midwives have no voice in healthcare institutions – or organisational influence and autonomy – nor in health policy
- The social and economic position and status of nurses and midwives are low. Salaries in private primary care practices and in public health centres and public hospitals are low
- There is no systematic workforce planning for nursing and midwifery. Current education and roles in practice (see below) are not tuned to the future roles of nurses and midwives in a person-centred, integrated health system

### Service delivery/practice: nursing practice in primary care

Individual (single-handed) GPs are contracted to the Health Insurance Fund, although several may work in the same health centre, from which they rent their premises. GP practices are obliged to employ a nurse but do not have administrative personnel. GP practices often lack adequate rooms and facilities to deliver modern, preventive, collaborative primary care and teamwork is limited.

North Macedonia has a nation-wide eHealth system, originally designed to facilitate making appointments in the hospital sector, called ‘Moj Termin’ (My Appointment). This system is centrally directed and has several applications, such as referrals and prescriptions. However, in primary care, consultations are not by appointment and double administration is present (WHO Regional Office for Europe, 2019). Not using an appointment system makes it difficult to organise the work of GPs and nurses in a way that justifies the independent roles of both and makes work planning in general difficult.

Primary care nurses have a limited, if any, role in prevention and health promotion. For example, they do not have separate consultations with patients with chronic conditions to enhance and support their self-management and work towards secondary prevention.

The primary care nurses spend most of their time managing the electronic and paper records concerned with targets (which are the basis for remuneration along with a capitation fee), checking prescriptions, referrals and patient registration. The administrative burden is increased by the duplication of paper and electronic records and records needed by different organisations. Hence, primary care nurses mainly perform administrative tasks to the detriment of clinical nursing (see Box 4). Nurses are not able to work to their full potential as independent professionals in primary care; consequently, current practice in primary care nursing deviates from the core elements of people-centred and integrated care (WHO, 2018). However, the potential contribution of nursing to a quality PHC service is recognised by the MoH (2019) white paper.

Box 4. Summary of PHC Nursing Practice Challenges

- Administrative burden created by the duplication of paper and electronic records and records needed by different organisations
- Administration undertaken by clinically qualified nurses who are therefore prevented from undertaking clinical work due to the amount of administration
- Lack of adequate rooms and facilities for PHC practitioners to deliver modern, preventive, collaborative PHC
- Lack of a PHC Nursing role with competencies, education and ongoing CPD
- Lack of a career pathway in PHC Nursing which would provide for Practice Nurses, Advanced Practice Nurses and Nurse Practitioners who could work alongside GPs
- Limitations on the autonomy of GPs (initial qualified doctors)

## Developments

**Figure 2.**
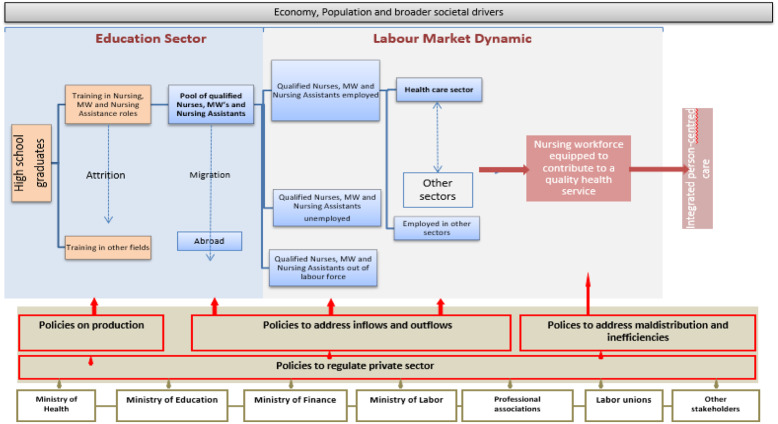
Summary of nursing workforce development issues. (*Source:* Adapted from: Global strategy on human resources for health: Workforce 2030. Fig. 2: Policy levers to shape health labour markets. WHO, Geneva 2016b: 13).

It was concluded from the information collected that nurses in the country had huge potential and there was a general recognition and enthusiasm to support the better contribution of nurses to achieving the aims of the North Macedonian PC strategy (MoH, 2019). Our analysis was organised into three areas: education, structural position and service delivery/practice, and a fourth area, workforce planning, was added as this underpins the other three areas. A Roadmap entitled *Making Change Happen: The Nursing and Midwifery Development Roadmap* (Groenewegen *et al.*, 2019b) was produced outlining the areas that needed to be addressed over a 10-year period to support the more effective contribution of nurses to meeting the healthcare objectives in the country. These actions are summarised in the adaptation of Fig. 1 to the situation of nursing and midwifery in North Macedonia in Fig. 2.

## Development 1. 10-year plan: Making Change Happen – the Nursing and Midwifery Development Roadmap

One of the first supportive actions identified in the Roadmap was the need for a national group to lead the development of PHC nursing in the country. The National Working Group for Moving Primary Care Nursing Forward in North Macedonia was launched during the second visit (November 2019). It is led by a nurse lead in the MoH and includes all important stakeholders.

## Development 2. Development of the pilot for a new model of PHC

To support the implementation of the new model of PHC in pilot test sites, the role of nurses and the scope of their work in PHC had to be developed (MoH, 2019). The pilot sites comprise of two municipalities in Skopje (the capital of North Macedonia). Each municipality has a large health centre/polyclinic which accommodate public health services including the vaccination service, patronage nurses, preventive and treatment dental care, laboratory facilities and general practices. The general practices, which comprise largely of a GP and a nurse, are private businesses renting space from the health centres. The pilot sites also include services located elsewhere in each municipality outside the health centres (home care nursing, stand-alone GP practices and other healthcare services).

During meetings with nurses, primary care doctors and managers in the two pilot health centres in January 2020 information was gathered on facilitators and barriers to change. Facilitators included the interest among some GPs in working in group practices, for nurses to undertake more clinical work and for there to be more teamwork between stand-alone GPs working in the wider community and those in the health centres. Barriers included the duplication of administrative work, the limited space in the health centres and the lack of appointment systems in primary care.

One of the main areas that was identified as needing development was the upskilling of nurses working in PHC. It was agreed that a train-the-trainers programme would be developed and delivered in June 2020 in Skopje, and the piloting of the new model of PHC in the two health centres was planned to start later in that year. However, due to the COVID-19 pandemic, revisions were made to these plans.

In consultation with the WHO Country Office and the National Nursing Working Group, the focus moved from a train-the-trainers model to a programme of online learning, available directly to the PHC nurses all over the country through an online platform. This decision drew on experience of the WHO Country Office with online modules for healthcare practitioners concerning COVID-19.

## Development 3. Online teaching course

**Figure 3.**
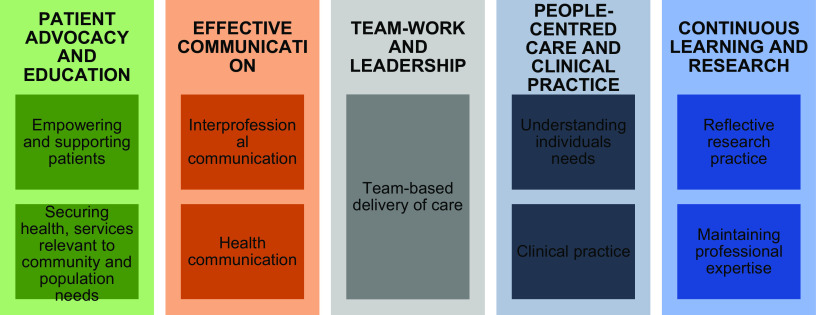
Competencies for nurses working in primary health care (WHO Europe, 2020: 3).

The plans for the programme were developed between the National Nursing Group, the WHO Country Office, the WHO Project Manager in North Macedonia and three of the authors (MSM, CS, RB), based on the WHO Office for Europe competency document (WHO Office for Europe, 2020; Atanasova and Tawilah, 2021). The competencies are grouped into five areas shown in Fig. 3.

The involvement of nurses was organised through a Nurse Facilitators Group that – apart from nurses – also included a FM educationalist.

The e-learning programme was called the Professional Development Programme for Primary Care Nurses and had the following aim and objectives:

1. **Aim:** to develop the knowledge and skills of PHC nurses and midwives to enable them to contribute most effectively to the delivery of the new model of PHC in North Macedonia

**Objectives:**

1. To develop the participants basic skills to enable them to contribute most effectively to the new model of PHC.
2. To enable PHC nurses and midwives to respond and put in place systems in PHC to manage the current and future epidemics.
3. To develop participants’ knowledge and skills in the management of common chronic conditions.
4. To develop the confidence of the participants to work as equal members of the PHC team.
5. To provide a means for participants to access continuing professional development (CPD) as part of their ongoing professional development.

The programme comprised a number of modules and between June 2020 and March 2021 there were a number of discussions regarding the content of the programme with the final structure shown in Box 5.

Box 5. Professional Development Programme for PHC Nurses

**Programme Structure**

- Module 1: Introduction to the programme
- Module 2: Foundations of Communication and Professionalism
- Module 3: Primary Care Nursing and COVID-19
- Module 4: Public Health
- Module 5: Health Education
- Module 6: Empowerment of Nurses and Midwives

Two of the authors (MSM and RB) identified the material to be included in the modules. Collaboration with colleagues from Coventry University (UK) provided access to learning resources. Their material on communication was incorporated into the Communication and Professionalism module. Development of the modules was also informed by discussions with the World Continuing Education Alliance Platform (https://wcea.education) and a review of modules on this platform.

Outline proposals were developed for each of the modules, and these were reviewed by the Nurse Facilitators Group. The structure of each module comprised of a presentation video followed by a workshop. Presentations for each module were developed and shared with the Facilitators Group. Once they had reviewed the presentations, these were translated into Macedonian. Scripts were developed for the modules and these were recorded in English on the translated presentations. The modules were then uploaded onto the Moj Termin (e-Health) platform, and information about the modules was disseminated by the WHO Project Manager to PHC and other nurses and the MoH. The nurses could undertake the modules in any order. For each workshop, a case study activity was developed, supported by a guidance document for the facilitators and a meeting prior to each workshop to review the activity and to make adjustments.

The structure of each module was similar, starting with the presentation outlining the aim and objectives of the module, reference to the relevant section of the WHO Office for Europe (2020) competencies and then content on the topic (see, e.g., Box 6). Throughout each presentation, there were activities and questions for the nurses to complete as they worked through the module as well as links to additional reading and resources. Each workshop started with a plenary outlining the work to be undertaken during the workshop. Participants then were split into small groups, each facilitated by one of the Nurse Facilitators, MSM or RB (who were supported by interpreters). The whole group then came back together for feedback. The workshops were recorded and made available on the e-Health platform.

Box 6. Definition of Empowerment Slide

**Definition of Empowerment**

In the Health Education Module, we identified empowerment of patients as a key element of health education

- How would you describe empowerment?
- What makes you feel empowered?
- Do you feel empowered in your workplace?
- How could you feel more empowered at work?

These are quite challenging questions but ones that we need to consider

Evaluation of the e-learning programme was undertaken in March 2021 (Bryar and Sánchez Martínez, 2021). Up to this time, 429 nurses had accessed the modules and 70–100 nurses had attended each of the workshops, indicating a high level of interest. Sixty-seven nurses completed the evaluation questionnaire within a short turn-around time of a week. Of these, 67, at least a third, had a university qualification, which suggests that the respondents may be a more highly educated group and, therefore, may not be representative of all the nurses registered on the programme. The responses received included problems, such as availability of computer equipment and stability of internet access, language issues and difficulties accessing links in the videos. Positive comments supported more blended learning and workshops, support for on-line learning and topics for further learning. The evaluation showed a high level of sharing of the content of the modules with a wide range of colleagues and team members, enthusiasm for the workshops and suggestions about increasing the frequency of workshops, reporting of application of learning to their practice and support for further CPD.

## Conclusion

The 10-year Roadmap: *Making Change Happen: The Nursing and Midwifery Development Roadmap* outlined the steps needed to develop an effective PHC and wider nursing and midwifery workforce in North Macedonia. Based on an analysis of the education, position in society and health care and the actual scope of practice of primary care nurses in North Macedonia, several developments were set in motion: establishment of the National Nursing Working Group, development of sites to pilot new ways of working in primary care, educational activities and actions to support this. The plans were overtaken by the COVID-19 pandemic and flexibly changed from a planned teach-the-teacher programme, focused on the pilot sites, into an e-learning programme for all nurses. A benefit of e-learning is that many more people can attend from a much wider geographical area than if this was a face-to-face programme. As primary care reforms are implemented throughout the country, this education will contribute to the nurses fulfilling their full potential as autonomous practitioners in primary healthcare teams.

The developments, described in this paper, can be used by other countries with similar challenges relating to the situation of nursing. The restricted scope of practice and the focus on (administratively) supporting doctors, instead of supporting patients, are not unique to North Macedonia.
